# The Use of Longitudinal ^18^F-FET MicroPET Imaging to Evaluate Response to Irinotecan in Orthotopic Human Glioblastoma Multiforme Xenografts

**DOI:** 10.1371/journal.pone.0100009

**Published:** 2014-06-11

**Authors:** Mette K. Nedergaard, Karina Kristoffersen, Signe R. Michaelsen, Jacob Madsen, Hans S. Poulsen, Marie-Thérése Stockhausen, Ulrik Lassen, Andreas Kjaer

**Affiliations:** 1 Department of Clinical Physiology, Nuclear Medicine & PET and Cluster for Molecular Imaging, Rigshospitalet and University of Copenhagen, Copenhagen, Denmark; 2 Department of Radiation Biology, The Finsen Center, Rigshospitalet, Copenhagen, Denmark; 3 Phase 1 Unit, Department of Oncology, The Finsen Center, Rigshospitalet, Copenhagen, Denmark; Wake Forest University, School of Medicine, United States of America

## Abstract

**Objectives:**

Brain tumor imaging is challenging. Although ^18^F-FET PET is widely used in the clinic, the value of ^18^F-FET MicroPET to evaluate brain tumors in xenograft has not been assessed to date. The aim of this study therefore was to evaluate the performance of *in vivo* ^18^F-FET MicroPET in detecting a treatment response in xenografts. In addition, the correlations between the ^18^F-FET tumor accumulation and the gene expression of Ki67 and the amino acid transporters LAT1 and LAT2 were investigated. Furthermore, Ki67, LAT1 and LAT2 gene expression in xenograft and archival patient tumors was compared.

**Methods:**

Human GBM cells were injected orthotopically in nude mice and ^18^F-FET uptake was followed by weekly MicroPET/CT. When tumor take was observed, mice were treated with CPT-11 or saline weekly. After two weeks of treatment the brain tumors were isolated and quantitative polymerase chain reaction were performed on the xenograft tumors and in parallel on archival patient tumor specimens.

**Results:**

The relative tumor-to-brain (T/B) ratio of SUV_max_ was significantly lower after one week (123±6%, n = 7 vs. 147±6%, n = 7; p = 0.018) and after two weeks (142±8%, n = 5 vs. 204±27%, n = 4; p = 0.047) in the CPT-11 group compared with the control group. Strong negative correlations between SUV_max_ T/B ratio and LAT1 (r = −0.62, p = 0.04) and LAT2 (r = −0.67, p = 0.02) were observed. In addition, a strong positive correlation between LAT1 and Ki67 was detected in xenografts. Furthermore, a 1.6 fold higher expression of LAT1 and a 23 fold higher expression of LAT2 were observed in patient specimens compared to xenografts.

**Conclusions:**

^18^F-FET MicroPET can be used to detect a treatment response to CPT-11 in GBM xenografts. The strong negative correlation between SUV_max_ T/B ratio and LAT1/LAT2 indicates an export transport function. We suggest that ^18^F-FET PET may be used for detection of early treatment response in patients.

## Introduction

The majority of primary brain tumors are gliomas and glioblastoma multiforme (GBM) is the most common and aggressive type of glioma in adults. The prognosis for patients diagnosed with GBM remains mainly palliative despite multimodal therapies, including aggressive surgery and radiotherapy combined with chemotherapy. The new Response Assesment in Neuro-Oncology (RANO) criteria was recently published [1]; however, there are still difficulties in assessing true tumor response on magnetic resonance imaging (MRI). Contrast enhancing and non-enhancing regions are non-tumor-specific and are influenced by different processes, such as subacute radiation effects, postoperative changes, changes in glucocorticoid dosage as well as anti-angiogenic treatments that affect the permeability of the tumor vasculature [1], [2]. One further challenge to the traditional, morphological imaging techniques is the wish to differentiate between responders and non-responders in the early phases of a treatment course.

Functional tumor imaging with positron emission tomography (PET) plays an increasingly important role in the diagnosis of cancer and monitoring of cancer therapy. Accordingly, PET with 2′-deoxy-2′-^18^F-fluoro-D-glucose (^18^F-FDG) have become a key imaging modality in the clinical management of a majority of cancer patients [3]. Due to the high rate of glucose metabolism in normal brain tissue and increased glucose uptake in inflammatory cells, ^18^F-FDG PET has shown diagnostic limitations when used for brain tumor imaging [4]. By contrast, radiolabeled amino acids have a relatively low uptake in normal brain tissue and usually accumulate intensely in tumor cells. The high tumor-to-brain (T/B) ratio makes radiolabeled amino acids particularly applicable in neuro-oncology *(5)*. A number of studies have demonstrated that O-(2-^18^F-fluoroethyl)-L-tyrosine (^18^F-FET) PET compared to MRI alone adds additional information about brain tumor growth [5]–[7]. Accumulation of ^18^F-FET in brain tumor cells is presumable linked to high expression of the L-type amino acid transporters (LATs), which are the major transport system for large neutral amino acid [8], [9]. Four subtypes of LATs have been identified of which subtype 1 (LAT1) and subtype 2 (LAT2) have been related to the cellular uptake of ^18^F-FET in cancer cells [10], although it has been speculated that ^18^F-FET accumulation primarily is mediated by LAT2 [11]. Despite that ^18^F-FET PET is widely used in the clinic, only a few animal studies have evaluated the performance of ^18^F-FET MicroPET in GBM xenografts [12]–[14]. Furthermore, the transport mechanisms of ^18^F-FET have not been thoroughly investigated [9], [15].

The primary objective of this preclinical study was therefore to evaluate the performance of ^18^F-FET MicroPET in monitoring brain tumor growth and in assessing a treatment response in an orthotopic xenograft model of human GBM. In addition, we wanted to test the hypothesis that ^18^F-FET accumulation was correlated to the gene expression of LAT1 and/or LAT2 in the tumor. Finally, we wanted to investigate the gene expression of Ki67, LAT1 and LAT2 in tumor specimens from GBM patients and compare it with the results from the xenograft tumors.

## Materials and Methods

### Ethics Statement

This study was performed according to the Declaration of Helsinki and Danish legislation. The use of patient tissue was approved by the Scientific Ethical Committee for Copenhagen and Frederiksberg (KF-01-327718) and permissions were given from the Danish Data Protection Agency (2006-41-6979). Written informed consent was obtained from the patients. Animal care and all experimental procedures were performed under the approval of the Danish Animal Welfare Council (2013-15-2934-00064).

### Establishment of the Human Orthotopic GBM Model

Establishment, maintenance and characterization of the neurosphere cell culture (NGBM_CPH048p6) used in this study has previously been described [16], [17]. Ten to 12 weeks old NMRI (Naval Medical Research Institute) nude female mice acquired from Taconic Europe (Lille Skensved, Denmark) were anaesthetized with Hypnorm/Midazolam (1 ml/100 g body weight) and the head was fixed in a stereotactic frame (KOPF model 963, 926-B and 922: Better Hospital Equipment Corp). A longitudinal incision was made in the scalp exposing the *calvarium*. Using a micro-drill, a burr-hole was drilled in the skull 1.5 mm right of the *sutura saggitalis* and 0.5 mm posterior to the *bregma*. Ten µl cell suspension (100,000 cells) of NGBM_CHP048p6 neurosphere cells was injected at a depth of 2–2.5 mm at a rate of 60 nl/sec using a 100 µl syringe with a 25-gauge needle (SGE100RN: World Precision Instruments, UK) placed in a micro infusion pump (Micro 4 pump and MicroSyringePump Controller: World Precision Instruments and KOPF model 1770-C: Better Hospital Equipment Corp). When injection was finished the needle was withdrawn after 1 min. Bupivacain (0.2 mg/100 g body weight) and Lidocain (1 mg/100 g body weight) were administrated in the incision site for local anesthetic before the skin was closed with an Ethicon 5-0 prolene suture.

### Experimental Design

Mice were injected with NGBM_CPH048p6 neurosphere cells at week 0 and the *in vivo* uptake of ^18^F-FET was monitored by weekly MicroPET and computed tomography (CT) scans to follow tumor growth. At confirmed tumor take, mice were divided in two groups and treated weekly with irinotecan hydroclorid (CPT-11) intraperitoneally (i.p.) (66.7 mg/kg) or 0.9% NaCl solution i.p. (control). Anti-cancer activity of CPT-11 in orthotopic glioma xenografts has been reported previously and the treatment regimen was based on these studies [18], [19]. At tumor take the treatment response was monitored by MicroPET/CT for two weeks and treatments were given the day after the scans were performed. In order to obtain similar tumor growth characteristic in the treatment and the control group, only mice with tumor take before 12 weeks were included in the study. In addition, the treatment and the control groups were matched according to standardized uptake values (SUV_max)_ and time to tumor take. Mice were humanly euthanized after two weeks of treatment, or if they showed tumor related symptoms such as neurological signs and/or considerable weight loss. Subsequently, the brains were removed and the tumor was isolated for RNA analysis. Two separate mice with confirmed tumor take were used to perform a dynamic ^18^F-FET MicroPET/CT and they were not included in the treatment part of the study.

### Synthesis of ^18^F-FET

^18^F-FET was synthesized using (2S)-O-(2-Tosyloxyethyl)-N-trityl-L-tyrosine-*tert*-butyl ester as precursor and synthesized on a GE TracerLab MX Synthesizer. All reagents and FET cassettes were purchased from ABX (Radeberg, Germany). The radiochemical purity was determined after measuring the content of fluoride-18 and other radioactive impurities in the ^18^F-FET solution measured with TLC and HPLC, respectively. The content of ethanol and acetonitrile was determined by GC analysis. The pH was measured with a pH-meter. In separate preparations the stability of the preparations was examined after 8 hours. HPLC was performed on a Dionex HPLC system (Dionex A/S, Denmark) equipped with an in-line radioactivity detector. The HPLC column was a Kinetx 2.6 µ, C18, 100A, 50×4.6 mm (Phenomenex, Denmark). The eluent was 98% 25 nM acetate buffer/2% acetonitrile pH 4.75 and a flow rate of 1.5 ml/min with UV detection at 275 nm. TLC plates were obtained from Merck and acetonitrile/acetate buffer pH 3.8 (70/30) was used as eluent. Residual solvents were determined on a Shimatzu GC 2014 (Holm & Halby, A/S, Denmark) equipped with a Chromosorb 101, 100–120 Mesh, 1/8″×10′ column, FID detector and helium carrier gas. The radiochemical purity of ^18^F-FET was >98% with a specific radioactivity ranging from 150–300 GBq/µmol at end of synthesis (EOS). The ethanol content was in the range 2.5–3.5% and the amount of acetonitrile was below the detection limit. The pH was 7.0–7.8. The radiochemical purity, ethanol content and pH did not change after 8 hours of storage at room temperature.

### MicroPET/CT Imaging

Mice were anaesthetized with Hypnorm/Midazolam (1 ml/100 g) and injected intravenously (i.v.) on average with 10.5±0.09 MBq ^18^F-FET. Mice were kept on a heat-pad to prevent hypothermia while anaesthetized and a 10 min static PET image was obtained at 20–30 minutes after tracer injection using a MicroPET Focus 120 (Siemens Medical Solutions, Malvern, PA, USA). The dynamic PET image was obtained two min before tracer injection and for 90 minutes. The energy window for the emission scan was set to 350–650 keV with a time resolution of 6 ns. PET data were post-processed into sinograms and subsequently reconstructed with the maximum a posteriori (MAP) reconstruction algorithm. Evaluation of the dynamic acquisition involved 18 time frames (18×5 min). The quantification unit was provided in Bq/ml. The intrinsic PET resolution was 1.2 mm full-width at half-maximum and the voxel size was 0.3×0.3×0.8 mm^3^. Scatter and attenuation correction were not applied [20]. A 4 minutes MicroCT scan was acquired in order to get anatomical information for brain delineation (MicroCAT II system, Siemens Medical Solutions). MicroPET and MicroCT images were manually fused using the Inveon software (Siemens Medical Solutions). A 3D spheric region of interest (ROI) was placed at the location of maximum tracer uptake in the tumor (ROI_T_). In the contralateral normal hemisphere a 4 mm^3^ spheric ROI was drawn (ROI_B_). To quantify the ^18^F-FET uptake, the standardized uptake values (SUVs) were calculated from the equation: SUV = C_T_/(D_inj_×W), where C_T_ is the radioactivity in tissue with the unit Bq/ml, D_inj_ is the injected dose and W is the weight of the mouse in grams. SUV_max_ was calculated from the voxel with the highest tracer concentration in the ROI. SUV_mean_ was calculated as the mean radioactivity in the ROI. Tracer uptake was expressed as T/B ratio of SUV_max_ (SUV_max_ ROI_T_/SUV_max_ ROI_B_) and SUV_mean_ (SUV_max_ ROI_T_/SUV_mean_ ROI_B_). We chose to express tracer uptake as a T/B ratio instead of absolute SUVs as there is a high unexplained inter-subject variability of SUV in the tumor model and also in GBM patients it is common to use the T/B ratio. Tumor take was predefined as a T/B ratio of SUV_max_≥1.3.

### Patient Specimens

Tumor specimens from 19 GBM patients obtained at primary surgery were randomly chosen and used for the gene expression analysis. The patient tumor (GBM_CPH048) used for establishment of the neurosphere cell culture NGBM_CPH048p6 utilized in the xenograft model was included. Isolated RNA from archival human jejunum was used as a positive control in the gene expression analysis as a high expression of LAT2 has been detected in the intestine [21].

### RNA Extraction and Reverse Transcription

After resection, tumor specimens from patients were snap-frozen and stored in liquid nitrogen. Total RNA was isolated using Trizol reagens (Gibco BRL 15596-018) and Qiagen TissueLyser before RNA purification with the RNeasy Miniki (Qiagen, Denmark). Resected xenograft tumors were immediately placed in tubes containing RNAlater (Sigma-Aldrich A/S, Denmark) and stored at 4°C for 2–3 days. Subsequently, the supernatant was removed and samples were stored at −80°C until further processing. The xenograft brain tumors were lyzed and homogenized in PrecellysR-24 (Bertin Techmologies, France). Total RNA from xenograft tumors was isolated with RNAzolRT in accordance with the protocol of the manufacturer (Molecular Research Center Inc., USA). The Agilent 2100 Bioanalyzer in conjunction with the Agilent RNA 6000 Nano Kits (Angilent Technologies Denmark A/S, Denmark) was used to measure the quality of the isolated RNA. RNA concentration was measured using the NanoDrop 1000 (Therme Fischer Scientific, USA). Total RNA (0.3 ug) was reversed transcribed (RT) using the AffinityScriptTM QPCR cDNA Synthesis Kit (Stratagene, USA) in accordance with the protocol of the manufacturer. RT reactions were performed using the Eppendorf Mastercycler Gradient (Eppendorf AG, Germany) and the protocol: incubation at 25°C for 5 minutes (primer annealing), 42°C for 15 minutes (cDNA synthesis) and 95°C for 5 minutes (termination of cDNA synthesis). Immediately after RT, samples were cooled and stored at −20°C.

### Quantitative Real–time PCR

The optimal housekeeping genes were selected from two panels of common endogenous control genes (TATA Biocenter, Sweden and the geNorm Kit, PrimerDesign, UK). The geNorm software was used to analyze gene expression stability and ubiquitin C (UBC) and actin beta (ACTB) were found to be the best candidate reference genes. Primers were designed using Beacon Designer™ (PREMIER Biosoft, USA). A BLAST search for sequence homology and a secondary structure search were included in the designs, and primers were optimized to be human specific and to distinguish between LAT1 and LAT2. Primer sequences were UBC-FP: 5′ctggaagatggtcgtacc-3′, UBC-RP: 5′gtcagggtcttcacgaag-3′, ACTB-FP: 5′-tggcatccacgaaactac-3′, ACTB-RP: 5′ggcagtgatctccttctg-3′, LAT1-FP: 5′-ggctgagttctggttcat-3′, LAT1-RP: 5′-tgtgtctgcctttcttgt-3′, LAT2-FP: 5′-ttgtcaggcagtggtagg3′, LAT2-RP: 5′-tggttctttgggtatgaatgtc-3′, Ki67-FP: 5′-tcccgcctgttttctttctgac-3′, Ki67-RP: 5′-ctctccaaggatgatgatgctttac-3′. All primers were purchased from Sigma-Aldrich (Sigma-Aldrich, USA).

The Brilliant SYBRGreen QPCR Master Mix (Stratagene) was used and gene expression was quantified on the Mx300P real-time PCR system (Stratagene). The following thermal profile was used: denaturation for 10 minutes at 95°C followed by 45 cycles of 30 seconds denaturation at 95°C, primer annealing for 1 minute at 60°C and 1 minute extension at 72°C. Subsequently, the PCR product was denatured for 1 minute at 95°C followed by a ramp down to 55°C and a dissociation curve was acquired by a stepwise increase in temperature from 55°C to 95°C with steps of 0.5°C/cycle. All samples were run in duplicates using 1 µl of cDNA and to each sample a no-template control (NTC) was included. No reverse transcription control (NoRT) for all samples was tested using the housekeeping genes and LAT1. All xenograft and patient samples were included in a single run for every gene and assays were optimized to have efficiencies between 90% and 110%. Quantification of results was based on the computation of target quantification cycle (Cq) values and housekeeping gene Cq values in the qbase^PLUS^ software (Biogazelle NV, Belgium) [22]. Genes of interest (GOI) were normalized to the arithmetic mean expression of the two housekeeping genes with a reference target stability of 0.66 (M-value) and 0.23 (CV-value). A default amplification efficacy of 100% was used. Results were reported as normalized relative quantities (NRQs). For relative comparison of the NRQs between murine and human samples a cDNA sample from human jejunum was included in all runs and GOI are expressed relative to the expression of GOI in the jejunum sample.

### Immunohistochemistry (IHC)

One brain from each treatment group was fixed for 24 hours in 4% paraformaldehyde (PFA), which subsequently was exchanged for EtOH 70%. After fixation the brains were divided in two by coronal cutting in the incision site and two pieces of each brain were embedded in the same paraffin block. From the brain anterior and posterior to the incision site, 4 µm histological sections were prepared for IHC. The sections were manually stained with hematoxylin and eosin (HE) for normal histological evaluation.

### Statistical Analysis

All statistical analysis was performed using GraphPad Prism version 5.01 for Windows (GraphPad Software Inc., USA). All data are presented as mean ± SEM (standard error of mean) if not stated otherwise. P<0.05 was considered statistically significant. In xenografts comparison between the treatment and the control group was performed using unpaired Student’s t-test. Univariate linear regression was performed in the gene expression analysis and the SUV_max_ T/B ratio was used. Comparison of the relative gene expression between patient and xenograft tumors was performed on log transformed data in order to obtain consistency with the Gaussian distribution. Student’s t-test with Welsh’s correction was used for the comparison between patient and xenograft tumors. All data were evaluated by the D’Agostino-Pearson normality test.

## Results

### Tumor Model Characteristics

**Figure 1:**
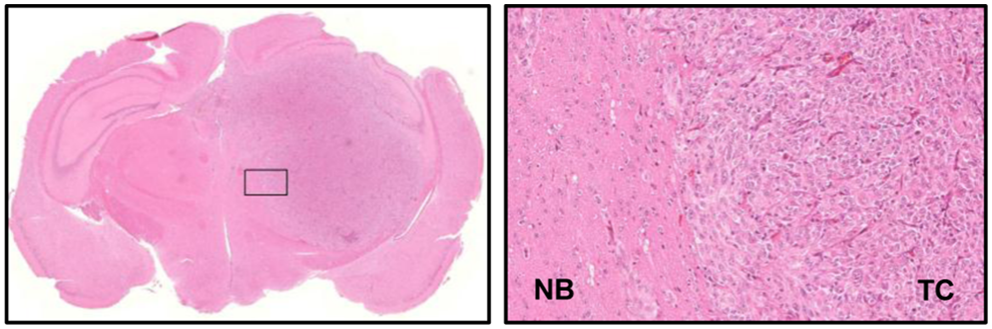
HE of xenograft tumor. A) HE section across a formalin-fixed paraffin-embedded mouse brain showing the GBM tumor 7 weeks after tumor cell injection. B) HE, magnification×20.

In order to establish GBM xenografts for the characterization of ^18^F-FET uptake, 20 mice were intracranially injected with NGBM_CPH048p6 neurosphere cells. Four mice were excluded from the study: Two mice due to absence of tumor take before the predefined limit of 12 weeks. One mouse was euthanized due to considerable weight loss, which was caused by intraventricular tumor growth, which was not visible on ^18^F-FET PET. The last mouse was excluded because of rapid tumor growth and weight loss within one week which hindered an evaluation MicroPET/CT. The tumor take rate (before 12 weeks) was 85% (17/20). Median time to tumor take was 6 weeks (range 3–11 weeks). A total of 16 mice were included in the treatment study: CPT-11 group (n = 8) and control group (n = 8). Figure 1 shows a HE stained section of a formalin-fixed paraffin-embedded mouse brain from the CPT-11 group 7 weeks after tumor cell injection. Marked cellularity and pleomorphism is evident in the section which are histopathological features typical for GBM.

### ^18^F-FET PET Imaging of Orthotopic GBM Xenografts

**Figure 2:**
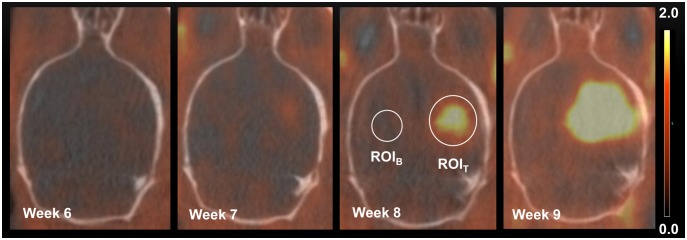
Fused ^18^F-FET PET/CT images. Fused ^18^F-FET MicroPET/CT images showing tumor progression 6–9 weeks after tumor cell injection. Transverse views through the brain of the same mouse. Illustrated in the figure is a ROI_T_ drawn round the region with maximum tracer uptake and a 4 mm^3^ ROI_B_ drawn in the contralateral hemisphere. Scale bar: 0.0–2.0 SUV_max_.

**Figure 3:**
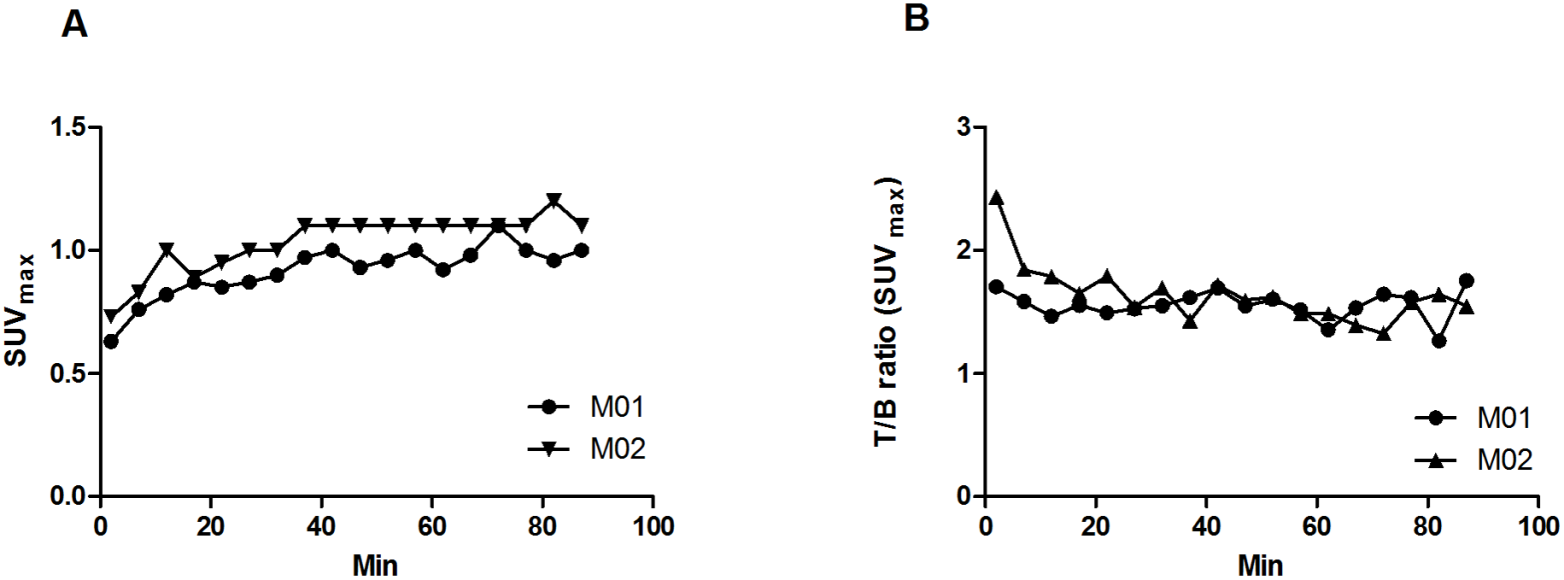
Time-activity curves of ^18^F-FET in xenografts. Time-activity curves of ^18^F-FET in two different mice (M01, M02) presented as SUV_max_ in the tumor ROI (A) and tumor-to-brain (T/B) ratio (B).

**Figure 4:**
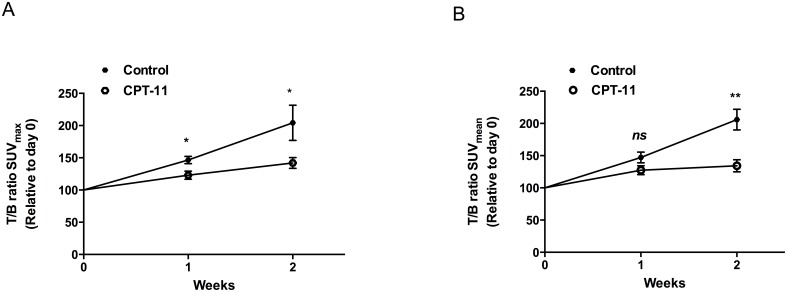
^18^F-FET uptake in xenografts. A) The relative T/B ratio of SUV_max_ versus time after tumor engraftment. B) The relative T/B ratio of SUV_mean_ versus time after tumor engraftment. Values expressed as mean ± SEM in the CPT-11 (n = 5–7) and in the control group (n = 4–7), *p<0.05 and **p<0.01.

Representative ^18^F-FET MicroPET/CT images of an orthotopic NGBM_CPH048p6 tumor from a single mouse are shown in Figure 2, with ROI_T_ and ROI_B_ illustrated. The ^18^F-PET images show a high ^18^F-FET uptake in the tumor and a very low background uptake in the brain. The size of the tumor and the intensity of the signal increased every week, indicating that ^18^F-FET MicroPET/CT can be used to monitor *in vivo* tumor growth. ^18^F-FET dynamics in the brain tumor and the contralateral normal hemisphere was evaluated in 2 separate mice not included in the treatment study. ^18^F-FET accumulation in the brain tumor was constantly increasing or stable (Figure 3A) and the T/B ratio was stable for the evaluation time (Figure 3B). In order to investigate whether the ^18^F-FET MicroPET/CT could be used to detect response to treatment, in this case CPT-11, the relative ^18^F-FET uptake in the two groups (the mean T/B ratio of SUV_max_ and SUV_mean,_ respectively) was plotted versus time after tumor engraftment (Figure 4A and 4B). The relative T/B ratio of SUV_max_ was significantly lower after one week (123±6%, n = 7 vs. 146±6%, n = 7; p = 0.018) and after two weeks (142±8, n = 5 vs. 204±27, n = 4; p = 0.047) in the CPT-11 group as compared with the control group. In addition, the relative T/B ratio of SUV_mean_ was significantly lower after two weeks (134±10%, n = 5 vs. 206±16%, n = 4; p = 0.0049) in the CPT-11 group, although after one week there was only a trend towards significance between the treatment and the control group (127±7%, n = 7 and 147±8%, n = 7; p = 0.09).

### Quantitative mRNA Expression of Ki-67, LAT1 and LAT2 in Xenografts

**Figure 5:**
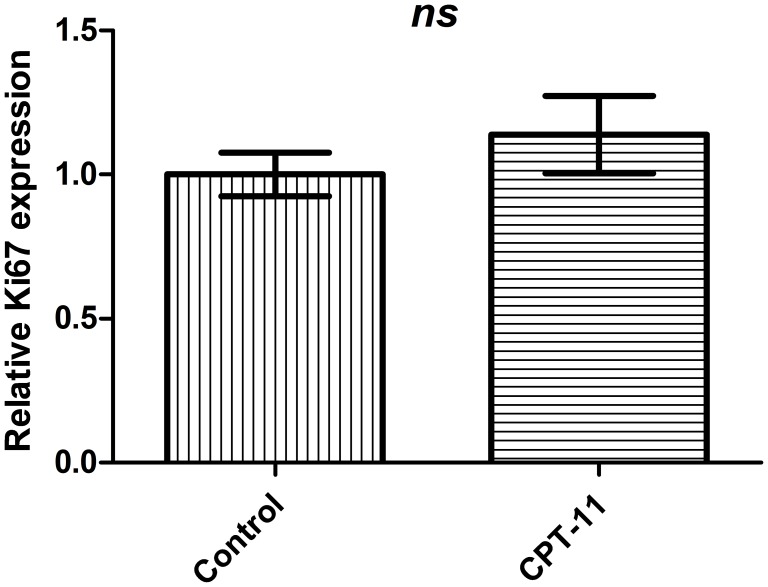
Ki67 expression in xenografts. The gene expression of Ki67 the CPT-11 (n = 7) relative to the control group (n = 4). Values expressed as mean ± SEM, *p* = 0.35.

**Figure 6:**
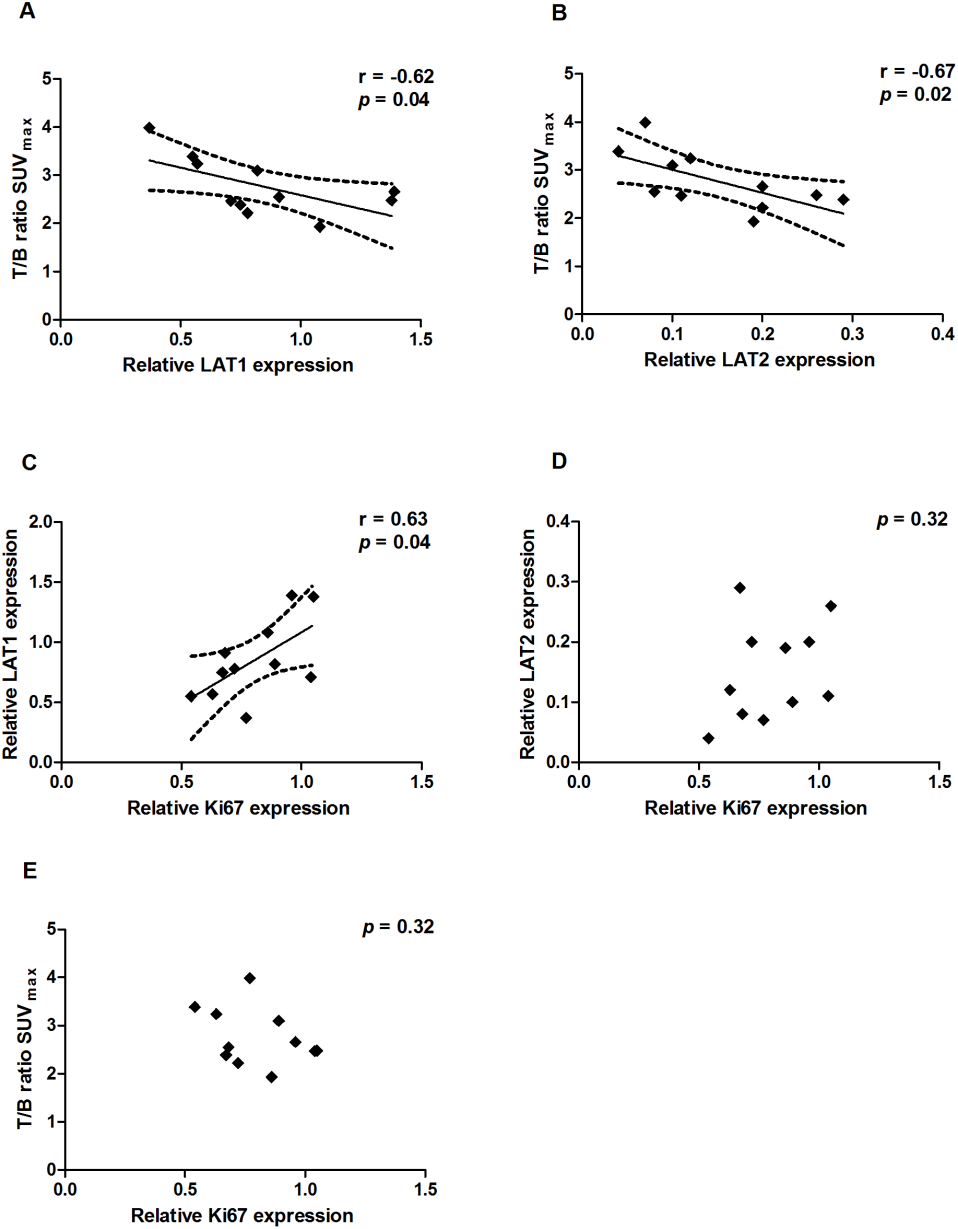
Gene expression and ^18^F-FET uptake in xenografts. Univariate linear regression analysis of gene expression (n = 11). A) LAT1 expression relative to T/B ratio of SUV_max_. B) LAT2 expression relative to T/B ratio of SUV_max_. C) Ki67 expression relative to LAT1. D) Ki67 expression relative to LAT2. E) Ki67 expression relative to T/B ratio of SUV_max_. The 95% CI is indicated by the broken lines.

As we were able to detect a tumor response to CPT-11 using ^18^F-FET MicroPET/CT we wanted to evaluate the effect of CPT-11 on tumor cell proliferation. For this, we compared the gene expression of Ki67 in the treatment group to the control group after two weeks of treatment. Surprisingly, we found no difference in the relative Ki67 expression in the treatment group as compared to the control group (1.14±0.1 vs. 1±0.08; *p* = 0.35), (Figure 5). To examine the relationship between the relative gene expression of the amino acid transporters LAT1 and LAT2 compared to the ^18^F-FET uptake, we performed qPCR against both transcripts and a univariate linear regression analysis. We found a strong negative correlation between the gene expression of LAT1 and the relative T/B ratio (r = −0.62, p = 0.04) as well as between the gene expression of LAT2 and the relative T/B ratio (r = −0.67, p = 0.02), (Figure 6A and 6B). Furthermore, we found a positive correlation between the gene expression of Ki67 and LAT1 (r = 0.63, p = 0.04), (Figure 6C). However, we did not find a correlation between the gene expression of Ki67 and LAT2 or between the T/B ratio and the gene expression of Ki67 (Figure 6D and 6E).

### Quantitative mRNA Expression in Xenografts Compared to Patient Specimens

**Figure 7:**
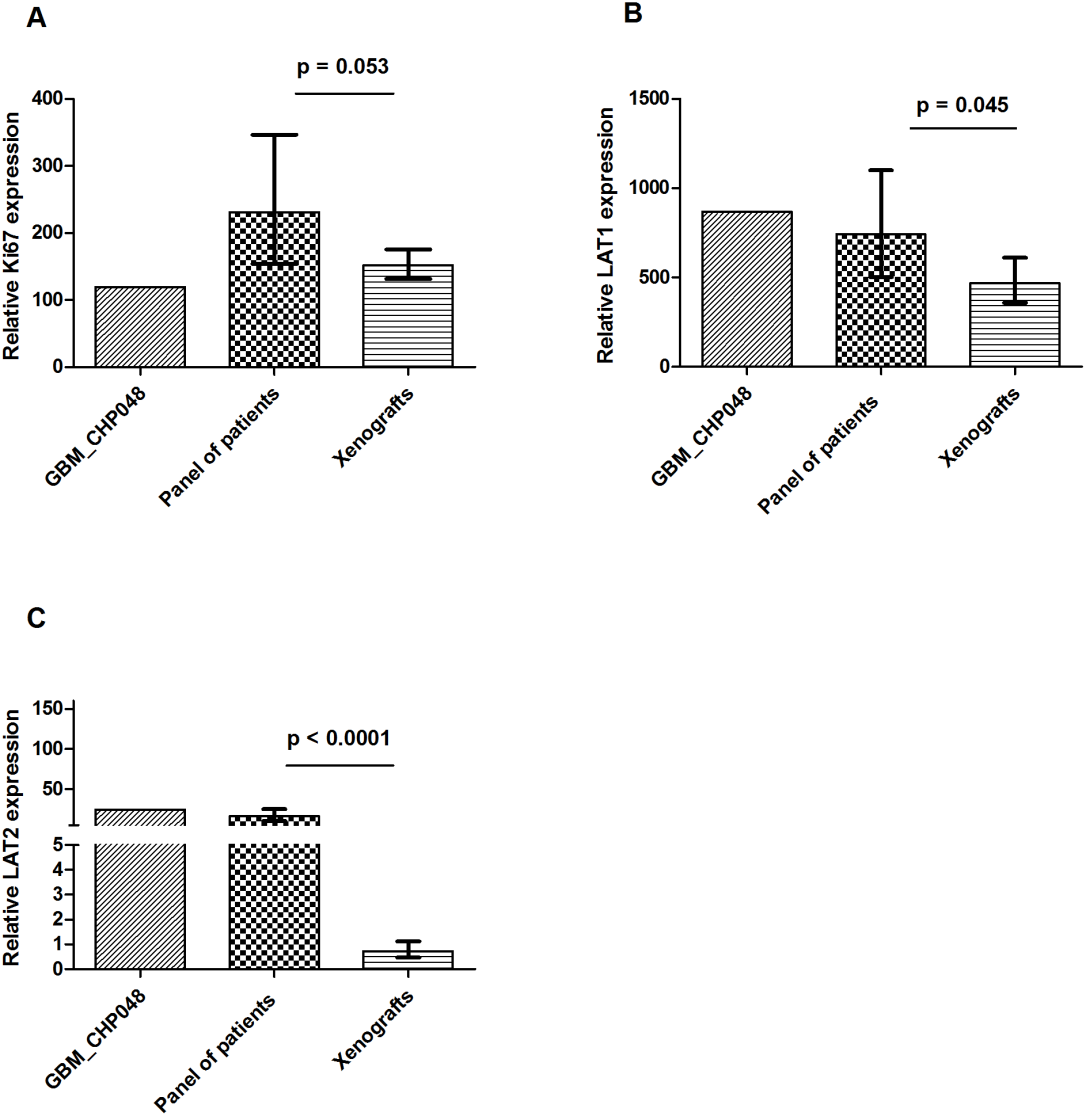
Gene expression in patients compared to xenografts. Relative gene expression in patient GBM_CPH048, the GBM patient panel (n = 19) and xenografts (n = 11). A) Ki67. B) LAT1. C) LAT2. All genes are normalized to housekeeping genes and are relative to human jejunum (jejunum = 100). Values are displayed as geometric mean±95% CI.

**Figure 8:**
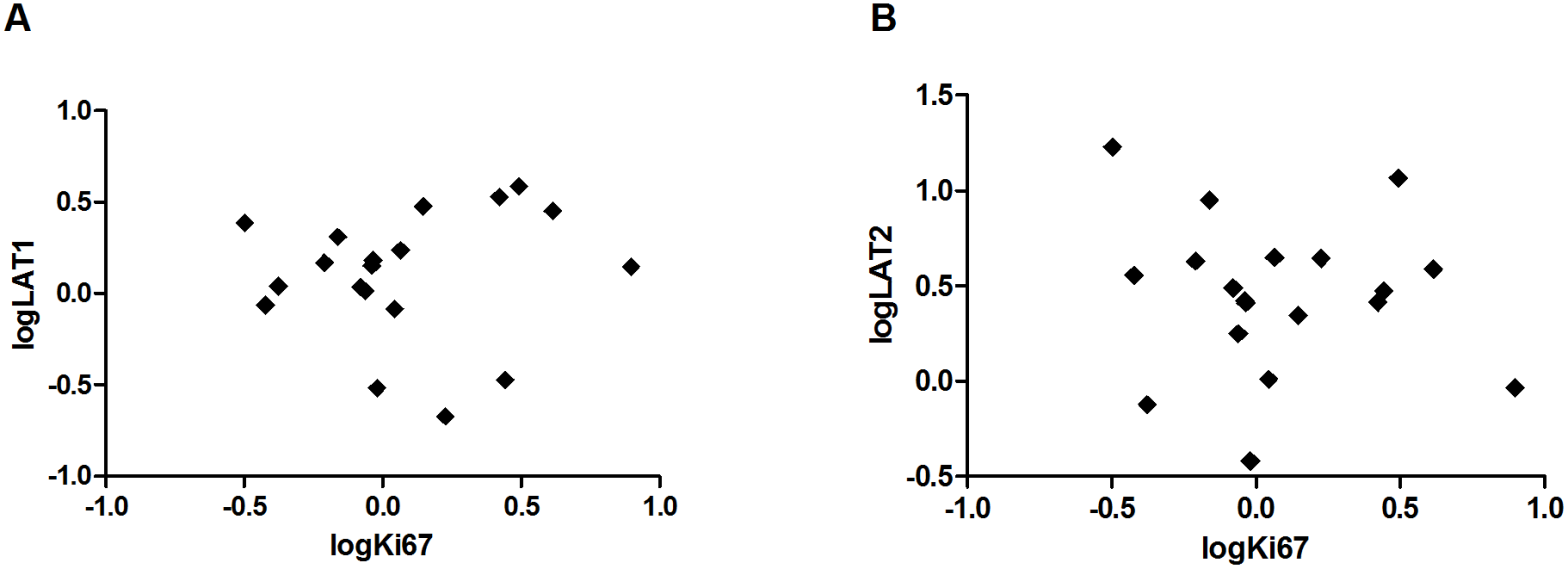
A) Person’s correlation between logki67 and logLAT1 in patient specimens. B) Pearson’s correlation between logki67 and logLAT2 in patient specimens (n = 19).

In order to investigate if the expression of Ki67, LAT1 and LAT2 were similar between the xenografts, the patient tumor (GBM_CHP048) used for the establishment of the xenografts and tumor specimens from a panel of 19 GBM patients, we performed qPCR and compared the NRQs relative to the gene expression in human jejunum, which was adjusted to 100. The relative gene expression of Ki67, LAT1 and LAT2 are illustrated in Figure 7. As expected, we found a larger variation in the human samples as compared to the xenograft tumors. The relative Ki67 expression was not significantly different between xenografts and patients (152; 95% CI: 131–176 vs. 231; 95% CI: 154–347; p = 0.053). However, the difference of the relative LAT1 expression was borderline significant with 1.6 fold higher LAT1 expression in GBM patients compared to the xenografts (744; 95% CI: 503–1099 vs. 467; 95% CI: 358–610; p = 0.045). In addition, we found a low LAT2 expression in the patients as compared to the expression of LAT2 in jejunum (16; 95% CI: 10–25 vs. 100). Surprisingly, the expression in xenografts was even lower as compared to the patients with an approximately 23 fold changes in relative expression of LAT2 (0.7; 95% CI: 0.5–1 vs. 16; 95% CI 10–25; p<0.0001). In general, the relative gene expression of Ki67, LAT1 and LAT2 in xenograft tumors were significantly different from the original patient tumor GBM_CPH048 (Figure 7). We did not find any correlations between Ki67 and LAT1 or LAT2 in the patient specimens (Figure 8).

## Discussion

In this study we demonstrated the feasibility of *in vivo* imaging of orthotopic human GBM in mice using ^18^F-FET MicroPET/CT. To our knowledge there are no other published reports evaluating the use of *longitudinal* ^18^F-FET imaging for treatment response evaluation in orthotopic glioma models. At present the most widely used amino acid tracers are L-methyl-^11^C-methionine (^11^C-MET) and ^18^F-FET [23]. Multiple clinical and preclinical studies have evaluated ^11^C-MET PET for the visualization of brain tumors, and it has been successfully used in neuro-oncology [9], [24]. However, in clinical practice, ^18^F-FET has logistic and economic advantages over ^11^C-MET due to the longer physical half-life of ^18^F (109.8 min vs. 20.4 min). ^18^F-FET is synthesized with a relatively high radiochemical yield (up to 40%), which is in contrast to other ^18^F labeled amino acids like ^18^F-fluro-L-tyrosine (^18^F-TYR) and ^18^F-fluro-L-methyl-tyrosine (^18^F-FMT) [25]. Only a few studies have evaluated the bio-distribution and tumor accumulation of ^18^F-FET in glioma models and these studies were, except from one study [12], all performed in rats [8], [13], [14]. The various advantages and disadvantages of the different glioma models are beyond the scope of this article. However, orthotopic tumor models are considered better predictive models of drug efficacy than traditional subcutaneous models [26]. With the use of orthotopic GBM models more advanced imaging techniques like MRI and PET are necessary. In the present study we have demonstrated the feasibility of using ^18^F-FET PET to monitor tumor growth non-invasive in a murine GBM model which makes is possible, in addition to MRI, to obtain complementary information about tumor growth. As such, the preclinical setting corresponds to the clinical setup were both imaging modalities frequently are used.

In addition to validation of ^18^F-FET as a feasible imaging tracer, it was possible to detect a treatment response with ^18^F-FET. Using the T/B ratio of SUV_max_ and SUV_mean_ we were able to distinguish between the treatment and the control group after 2 weeks of treatment. Furthermore, using the T/B ratio of SUV_max_ we detected a treatment response already after one week of treatment and results for the T/B ratio of SUV_mean_ were similar, although borderline significant. These results are in line with recently published results from the clinic, where it was possible to identify responders to bevacizumab and CPT-11 with ^18^F-FET PET at an early follow-up (median 4.9 weeks) [6]. In another clinical study, it was similarly concluded that changes in the ^18^F-FET PET signal might be a useful measure to predict treatment response at an early stage of GBM [7]. Our findings suggest that responders and non-responders may have been differentiated by ^18^F-FET PET at an even earlier time point.

In order to interpret ^18^F-FET, it is essential to understand the transport mechanisms and the major factors that influence the transport and tumor uptake of ^18^F-FET. The tumor uptake of ^18^F-FET is related to the higher transport rate of amino acids rather than to proliferation. In addition, a disruption of the blood-brain barrier (BBB) is not mandatory for ^18^F-FET uptake in gliomas [27]. As such, ^18^F-FET is different from the proliferation tracer 3′-deoxy-3′-^18^F-fluorothymidine (^18^F-FLT) which is a marker of DNA synthesis. ^18^F-FLT is not transported across the intact BBB which affects the sensitivity of ^18^F-FLT in gliomas [28]. The major transport systems for neutral amino acids like L-tyrosine are: System A (alanine preferring), system ASC (alanine-serine-cystine preferring) and system L (leucine preferring) [29]. A few *in vitro* studies have determined the Na^+^-independent system L as the main transport system of L-tyrosine and its analog ^18^F-FET [8], [9]. Among the four subtypes of system L, especially LAT1 expression has attracted much attention and it has been investigated in several cancer types, although only a few reports exist regarding LAT1 expression in gliomas [30]. In a clinical study the LAT1 IHC staining was located to the vascular endothelium as well as the tumor cell membrane and cytoplasm in tumor specimens from patients with glioma [30]. In a rat C6 glioma cell line, LAT1, but not LAT2, was expressed, and in normal astrocytes LAT2, but not LAT1, was expressed, indicating LAT1 as a possible target for anti-cancer therapy [15]. In the present study, we found LAT1 as well as LAT2 to be expressed in GBM tissue from patients and from xenografts. We also observed a positive correlation between LAT1 and Ki67 in the xenograft tumors which is in line with another study where LAT1 correlated with the glioma pathological grading, and the IHC staining of Ki67 [30]. However, and in contrary to this study [30], we failed to detect this LAT1/Ki67 correlation in our patient samples. Other reports have confirmed the expression of LAT1 at the blood-brain barrier (BBB) [21], however, expression of LAT2 in the BBB is controversial and limited information exists regarding LAT2 expression in gliomas [21]. In our study, we found a much lower expression of LAT2 in xenografts compared to patient specimens. As we did not perform IHC, we are unable to conclude if LAT1 and LAT2 were located primarily at the BBB, in the tumor cells or if the location is overlapping. If LAT2 primarily is located at the BBB, this could be a possible explanation for the low expression of LAT2 in xenografts as the LAT2 primers were specifically designed for human LAT2 and tumor vessels in the xenograft tumor are primarily murine. Different expression of LAT2 between species is another possible explanation for this difference in LAT2 expression. However, this needs further investigation.

As described above, the transport of ^18^F-FET is mainly facilitated by system L and presumably linked to the expression of LAT1 and/or LAT2 [10], [11], [31]. The dynamic ^18^F-FET PET performed in this study demonstrated accumulation and retention of ^18^F-FET in the normal brain and in the brain tumor. A similar pattern is seen in some GBM patients, while other GBM patients show a decreasing pattern with an early wash out of ^18^F-FET [32]. In the present study, we observed a strong negative correlation between the relative ^18^F-FET T/B ratio and the gene expression of LAT1 and LAT2, which could indicate an export transport function. The LATs are amino acid exchangers with 1∶1 stoichiometry and the net direction of ^18^F-FET depends on the extra- and intracellular concentrations of ^18^F-FET [21]. The retention mechanisms of ^18^F-FET have not been clarified and one could speculate that a saturation of the retention mechanism is possible in the xenograft model where ^18^F-FET is given in much higher concentrations compared to human patients. As such, ^18^F-FET would, to a small extent, be transported out of the cell as the retention system is saturated and the blood concentration is decreasing. This transport out of the cell could be dependent on the amount of LATs present in the cell membrane. As a result there would be a negative correlation between the T/B ratio and LAT1 and LAT2, although most of ^18^F-FET is still retained in the tumor cells as demonstrated in the dynamic ^18^F-FET PET (Figure 3). However, the small sample size of this study makes the observed correlations less reliable and it needs to be verified in larger studies.

In the present study, the relative difference in T/B ratio between the treatment and the control group was not reflected in a decrease in the Ki67 gene expression level in the treatment group. Although other studies have demonstrated anti-cancer activity of CPT-11 in GBM murine models [18], [19], we did not perform a survival analysis in this study and further studies are thus needed to explore if the changes in the ^18^F-FET uptake reflect true anti-cancer activity. The controversial topic about protein expression and mRNA level is another possible explanation for the observed unchanged Ki67 gene expression level in the present study. In general, expression of proteins correlate with their corresponding mRNAs, but the correlation is not very strong [33]. It remains questionable if small changes at the protein level are reflected in the gene expression level. The correlation between mRNA expression and protein level of Ki67 in this tumor model thus needs further investigation.

The optimal imaging strategy for evaluating patients with GBM has not been elucidated and comparative evidence whether PET has superior properties compared to modern MRI techniques or whether a specific PET tracer outperforms another is limited. Several clinical studies have documented the diagnostic performance of ^18^F-FET PET in primary brain tumors, and in conjugation with MRI, ^18^F-FET PET has revealed supplementary information on tumor growth and metabolism [6], [34]. Furthermore, a good correlation between ^18^F-FET uptake and treatment response has also been demonstrated in clinical studies [5]–[7], [35]. In the present study, we used a patient derived GBM cell line in a murine model and demonstrated the feasibility of monitoring a treatment response with ^18^F-FET PET. The development and implementation of new anti-GBM therapies require valid tumor models and a translational method for drug testing and response assessment. ^18^F-FET PET (in conjunction with MRI and/or bioluminescence) can possibly be used to evaluate new treatment regimens and novel therapeutic agents in several human xenograft GBM models with different molecular characteristic. With more accurate animal models and imaging techniques we will likely create better results that translate into satisfactory treatment outcomes in the clinic.

## Conclusion

In conclusion, we have demonstrated the feasibility of *in vivo* imaging of orthotopic human GBM in a murine model with ^18^F-FET PET. In addition, we found that with ^18^F-FET uptake we were able to detect a CPT-11 treatment response after one and two weeks of treatment, suggesting that ^18^F-FET uptake may be an early and non-invasive biomarker for detection of anti-tumor activity or treatment failure in preclinical and in clinical studies. As such, this study supports the additional use of ^18^F-FET PET in the evaluation of patients with GBM and in preclinical trials. We found a strong positive correlation between the gene expression of Ki67 and LAT1 in xenografts, however there was no correlation in patient specimens. Furthermore, we found a much higher expression of LAT2 in patient specimens compared to xenografts, which could be caused by human specific LAT primers or indicate a difference between species. Interestingly, we found a strong negative correlation between the T/B ratio and the gene expression of LAT1 and LAT2 in xenografts, which may be explained by the ^18^F-FET kinetics and tumor cell retention mechanisms. However, further studies are needed to clarify the ^18^F-FET dynamics and exact transport mechanisms in humans and in xenografts.
